# Graphene metasurface hits the point

**DOI:** 10.1038/s41377-023-01159-6

**Published:** 2023-05-08

**Authors:** Jiazheng Qin, Mengjia Wang, Cheng-Wei Qiu

**Affiliations:** Department of Electrical and Computer Engineering, National University of Singapore, Singapore, 117583 Singapore

**Keywords:** Metamaterials, Electronic properties and devices

## Abstract

Exceptional points pose exceptional difficulties to access and encircle. By simply gating graphene, it is now easier to hit the exceptional point.

Loss is not always the trouble-maker. They can be a helping hand, say, in non-Hermitian systems^1^. Interestingly, such systems host branching point degeneracy, known as exceptional points (EPs)^2^. At EPs, eigenvalues and eigenvectors coalesce simultaneously. Non-Hermitian degeneracy underpins many intriguing effects^3,4^, such as PT-symmetric phase transition, unusual Berry phase accumulation, as well as ultrahigh sensitivity to perturbations. These effects have recently been utilized in photonic designs to gain unconventional functionalities^5–8^, including asymmetric waveguiding, robust single-mode laser, coherent perfect absorption, omnipolarization converter, ultrasensitive sensors, etc.

In non-Hermitian research, it is of central importance to “pick out” EPs in the parameter space. Existing experimental studies heavily rely on the repeated fabrication and characterization of passive devices with varying geometric parameters. Due to the fabrication error and the high sensitivity of non-Hermitian systems near EPs, it is challenging to obtain a high accuracy of realizing EP conditions. Besides, such a passive and repetitive method sets limitations to the exploration of the intriguing phenomena related to the dynamic encirclement around EPs^9–11^. Alternatively, active devices have recently emerged as a more promising candidate for such studies^12,13^.

In this issue of *Light: Science & Applications*, Prof. Bumki Min, Prof. Teun-Teun Kim, and their team have introduced an electrical and spectral method for resolving chiral exceptional points (EPs) and elucidating the implications of chiral mode collapse in a non-Hermitian gated graphene metasurface^14^.

The authors constructed their graphene metasurface from an array of two coupled split-ring resonators (SRRs), which are orthogonally arranged with overlapping resonance but different scattering and radiation rates as shown in Fig. 1a. Moreover, a graphene microstrip is deposited on top to bridge each pair of SRRs. By manipulating the gate voltage (Fermi level) applied on the graphene, the inherent losses of the two SRRs are non-uniformly adjusted. The effective Hamiltonian of such an active polarization metasurface is a non-Hermitian Jones matrix, parametrized by the incident wavelength and the gated voltage. The authors employed time-domain spectroscopy and a broadband pulse to measure the complex eigenvalue of the transmission spectrum, from which the Jones Matrix can be obtained. By electrically tuning the gate voltage and spectrally resolving the incident frequency, a precise, real-time access to the parameter space can be realized with a single metasurface.

**Fig. 1:**
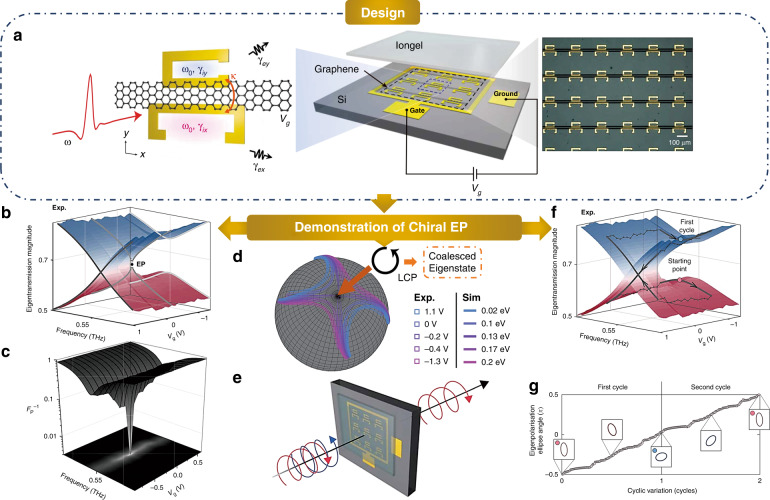
Experimental demonstration of the chiral EP with a non-Hermitian gated graphene metasurface. **a** Schematic rendering and microscopic image of the terahertz metasurface. **b** Measured eigentransmission magnitude. **c** Inverse plot of the Petermann factor. **d** Polarization eigenstate plotted on a Poincare ´sphere. **e** Counterintuitive polarization transmission at EP. **f** Cyclic encirclements around the chiral EP on the Riemann surface. **g** Dynamic evolution of the polarization eigenstate. The figures are adapted from the authors’ original publication

With this finely tuned design, Prof. Bumki Min, Prof. Teun-Teun Kim and other colleagues precisely mapped the eigenspace to capture the chiral EP. As shown in Fig. 1b, the measured eigen transmission exhibits a typical self-intersecting Reimann surface structure with a branch point denoting the EP. The EP manifests itself as a sharp dip in the inverse plot of the Petermann factor ($F_p^{ - 1}$, Fig. 1c). A value down to 3 × 10^−4^ at this dip indicates that the EP has been accurately identified.

The gated graphene metasurface allows the authors to investigate the abrupt mode coalescence and exotic polarization properties at EP. As illustrated in Fig. 1d, when the EP is approached, the paired eigenstates coalesce into a single left circular polarization (LCP) state located at the south pole. The missing eigenstate has been revealed by a counterintuitive finding: LCP transmission is totally cancelled when the input state is a specific combination of left and right circular polarizations (Fig. 1e). The authors also showed another interesting consequence of the mode collapsing. They found an asymmetric RCP-to-LCP conversion when light travels through the metasurface in opposite directions.

Finally, the dynamic behaviour of the system around EP was also studied. To this end, the authors tuned the system parameters to encircle the EP on the Riemann surface and monitored the orientation of the polarization eigenstate (Fig. 1f, g). The eigenpolarization swaps to the orthogonal state after 1-turn encirclement while returns to its original state after 2-turns. These results reveal a polarization vortex centred at the chiral EP with a half-integer topological charge.

This work showcases the potential of gated graphene metasurfaces as a tunable platform for non-Hermitian research and application. In future, more possibilities can be expected by extending such a compact and active design to higher parametric dimensions and other optical functionalities. For example, we can consider more sophisticated metasurfaces and gating designs to incorporate other controllable parameters to investigate the richer physics associated with higher-order EPs^15^. Additionally, the flexibility of tunable non-Hermitian devices can be used to develop advanced optical control and sensing applications.
